# Supplementary material for: A HAZOP-based hazard identification model for urban gas accidents: Development and empirical validation
Source: PLoS One. 2025 Oct 27;20(10):e0333431. doi: 10.1371/journal.pone.0333431 (PMC12558524; doi:10.1371/journal.pone.0333431)
Supplement: Attachment — Submitted filename: Response to Reviewers.docx [file pone.0333431.s001.docx]

Manuscript ID: PONE-D-25-41119

Title: A HAZOP-based hazard identification model for urban gas accidents: Development and empirical validation

Authors: Bin Tian et al.

Dear Editor and reviewers：

On behalf of all authors, we thank the Academic Editor and reviewers for their constructive comments and suggestions, which have helped us improve the quality and clarity of the manuscript. Below, we provide a point-by-point response to all comments. All changes in the manuscript are highlighted in the revised version with track changes.

Journal Requirements:

1. [PLOS ONE style]

Response: We have carefully reviewed the PLOS ONE formatting guidelines and reformatted the manuscript accordingly, including file naming, section headings, and reference style.

2. [ORCID]

Response: The corresponding author (Bin Tian) has verified his ORCID iD (https://orcid.org/0009-0001-3391-9158) in Editorial Manager.

3. [Code sharing]

Response: This study does not involve author-generated code. Therefore, this requirement does not apply.

4. [Data availability]

Response: All data underlying the findings of this study are fully available within the manuscript. As this is a methodological study based on a HAZOP-based hazard identification framework, no external data collection or computational simulations were conducted. The model application, including all relevant parameters, deviation analyses, and resulting hazard identifications, is fully described in the text. Therefore, the manuscript itself constitutes the complete minimal data set, and no additional data deposition is required.

5. [Role of funders]

Response: We have added the following statement in the cover letter: "The funders had no role in study design, data collection and analysis, decision to publish, or preparation of the manuscript."

6. [Funding in manuscript]

Response: All funding-related text has been removed from the manuscript. Funding information is now only included in the submission system.

7. [Table reference]

Response: We have added a reference to Table 1 in Section 2.4: " The main definitions are summarized below as Table 1."

8. [Citation recommendations]

Response: No specific citations were recommended by the reviewers. All references have been verified for accuracy and relevance.

9. [Reference list]

Response: The reference list has been checked and formatted according to PLOS ONE’s sequential numbering style. All citations are accurate, and no retracted papers are cited.

---

Reviewer #1:

Comment: The validation strategy could be further strengthened by involving subject matter experts and more external case studies to reduce bias.

Response: We fully agree with this insightful comment. In response, we have revised the subsection (4.2 Limitations of the Model), in which we explicitly acknowledge the limitation of single-case validation and emphasize the need for future multi-case and independent-team validation to enhance objectivity and generalizability. The following text has been added to Section 4.2(2):

"To better evaluate the model’s applicability and stability, future work should include multi-case comparative analyses conducted across diverse environments and ideally by independent teams. Such efforts would help minimize potential bias and strengthen the empirical foundation of the proposed framework [26,27]."

---

Reviewer #2:

Comment 1: Better highlight why HAZOP is superior compared to other methods (e.g., FMEA, bow-tie).

Response: We have added a comparative statement in the Introduction:

" Although several structured risk assessment methods—such as Failure Mode and Effects Analysis (FMEA), bow-tie analysis, and Fault Tree Analysis (FTA)—have been applied in safety engineering, each has limitations that constrain their effectiveness in complex urban gas systems [22]. FMEA primarily focuses on equipment failure modes and their consequences, but often neglects systemic issues related to human behavior, organizational processes, and environmental conditions. Bow-tie analysis and FTA, while effective in visualizing causal pathways and mitigation barriers, typically requires prior knowledge of initiating events and is less suited for proactively identifying unknown or unanticipated hazards. These approaches may therefore fall short in contexts where risks emerge from dynamic interactions across technical, human, and managerial domains. In contrast, Hazard and Operability Analysis (HAZOP) offers a more systematic and structured framework for hazard identification. By systematically applying guide words (e.g., “no,” “more,” “less,” “reverse”) to predefined parameters across multiple dimensions—such as human, machine, environment, and management—HAZOP enables a comprehensive examination of potential deviations, even in the absence of historical incident data. This deviation-driven approach supports proactive, thorough, and scenario-independent hazard detection, making it particularly well-suited for socio-technical systems like urban gas networks."

Comment 2: Inconsistent figure captions (e.g., “Fig.” vs “Figure”).

Response: All figure captions have been standardized to “Fig X.” format, in accordance with PLOS ONE guidelines.

Comment 3: References are not in order.

Response: The reference list has been re-ordered according to the sequence of citation in the text, following PLOS ONE’s numbering system.

Comment 4: Overuse of capitalization in headings.

Response: All section headings have been revised to sentence case (only first word capitalized), e.g., “Construction of the hazard identification model”.

Comment 5: Keywords not in alphabetical order.

Response: The keywords have been re-ordered alphabetically:

"Case study; Framework development; Hazard identification model; HAZOP; Urban gas accident"

Comment 6: Inconsistent number formatting in Introduction.

Response: All numerical values have been standardized:

- “94.394 million” → “94.39 million”
- “558.3287 million” → “558.33 million”
- Other values (e.g., 27.90%, 20.79 billion m³) were already appropriately formatted.

---

We believe that the revised manuscript has addressed all concerns and is now suitable for publication in PLOS ONE. Thank you again for the opportunity to improve our work.
